# Observational pilot study on the influence of an app-based self-management program on the quality of life of women with endometriosis

**DOI:** 10.1007/s00404-024-07468-4

**Published:** 2024-06-13

**Authors:** Nadine Rohloff, Markus Rothenhöfer, Teresa Götz, Sebastian Daniel Schäfer

**Affiliations:** 1 Endo Health GmbH, Theaterstraße 56, 09111 Chemnitz, Germany; 2 Department of Gynecology and Obstetrics, Clemenshospital Münster, Düesbergweg 124, 48153 Münster, Germany

**Keywords:** Endometriosis, App, Quality of life

## Abstract

**Purpose:**

Endometriosis can significantly impair the quality of life of those affected. Multimodal self-help measures are recommended but often difficult to access. Smartphone apps have been shown to improve the quality of life for other conditions with chronic pain. The aim of this study was to examine whether there is evidence of beneficial effects of the smartphone app “Endo-App^®^” and whether a multicenter randomized controlled trial should be planned to substantiate these effects.

**Methods:**

In a sample of *N* = 106 women affected by endometriosis the present study determined the influence of the use of Endo-App^®^ on their quality of life. Among others, the validated questionnaire *Endometriosis Health Profile* from Oxford University was used for this purpose.

**Results:**

The use of Endo-App^®^ lead to a highly significant improvement in quality of life already after 2 weeks. A statistically significant change was found for nine out of ten measured variables of quality of life. A series of further analyses validated that the measured positive effects were not due to other confounding factors.

**Conclusion:**

In summary, the results indicate that the quality of life of women with endometriosis improved by the digital self-management tool Endo-App^®^. More studies are needed to further explore the influence of the app on quality of life and as confirmatory evidence of beneficial effects. For this purpose, a randomized controlled trial should be conducted over a longer period of time.

**Trial registration:**

This trial is registered at clinicaltrials.gov under the registration number NCT05528601 on August 18, 2022. It was retrospectively registered.

## What does this study add to the clinical work

**Table:** 

The quality of life of endometriosis patients improved by the use of a digital self-management tool introduced in this study in patients with endometriosis. A prospective long-term multicenter randomized controlled trial is needed to further explore the effect of this patient-controlled health intervention.	

## Introduction

An estimated 1.7 million women in Germany suffer from endometriosis. Approximately 40,000 new diagnoses are made each year [1].

The disease severely limits the quality of life of those affected [2]. The leading symptoms include sterility, pain symptoms and accompanying functional complaints. Due to the long course of the disease, frequent recurrences even after surgery [3] and the lack of a curative therapy, the symptoms often become chronic [4]. Chronic pain has the strongest deteriorating impact on quality of life [5].

Experts from the German Pain Society agree that it is advisable to introduce patients with chronic pain of any etiology to measures with which they can help themselves [6]. Physical therapy, nutritional therapy, and informational approaches, as well as stress reduction and symptom diaries, are cited as universally applicable options [6].

The current S2k guideline on the treatment and diagnosis of endometriosis of German-speaking countries also emphasizes this. It is stated that patients should be informed about self-help services. The guideline also recommends complementary therapies with the goal of promoting skills in disease coping and self-management, support in behavioral and lifestyle changes, and maintaining earning capacity [7].

A multimodal, interdisciplinary approach to endometriosis therapy as an adjunct to standard therapy is recommended as it aims to significantly improve patients’ quality of life [8–11].

However, these measures are currently only accessible to a very limited proportion of patients and are rarely covered by health insurances.

For 2017, the health report of the Robert Koch Institute reports only 811 patients diagnosed with endometriosis in rehabilitation clinics [12]. Researchers, treating physicians, and patients therefore demand an improvement in patient care with the central goal of improving quality of life [9, 10]. Thus, in addition to causal therapy, supportive care and help for self-help are also necessary. Access to reliable and trustworthy information is currently limited as well [13, 14].

Most of the complementary therapies and self-management strategies mentioned above have to be carried out by the patients themselves in everyday life. The positive effect of self-care counselling for women with endometriosis has already been shown in a few randomised trials [15, 16]. For other conditions with chronic pain, it has already been shown that smartphone apps can support these self-care therapies at home, positively influencing pain, symptoms, and disease management [17]. Therefore, it can be expected that a mobile application via smartphone can be effective in improving the quality of life in endometriosis as well. Nonetheless, studies in this regard are still missing.

The Endo-App^®^ was developed as a medical device. The aim of this study was to investigate the app’s effect on quality of life. It was examined whether there is evidence of positive care effects and whether a multicenter randomized controlled trial is worthwhile.

## Materials and methods

### Endo-App

The Endo-App is a digital health application specifically designed to support the multimodal therapy of endometriosis. It provides evidence-based and guideline-compliant content, methodologies, and exercises rooted in multimodal pain therapy, helping users manage their condition. The app offers features like a detailed endometriosis diary, exercise guides, nutritional advice, educational articles and videos, psychosocial support, stress-reducing concepts and guidance on positive coping. It also educates users about the links between their symptoms and the medical reasons behind them. For tough times, there is an emergency plan to assist in managing severe pain. Overall, the app empowers users, giving them a sense of control over their condition.

### Sample

**Fig. 1:**
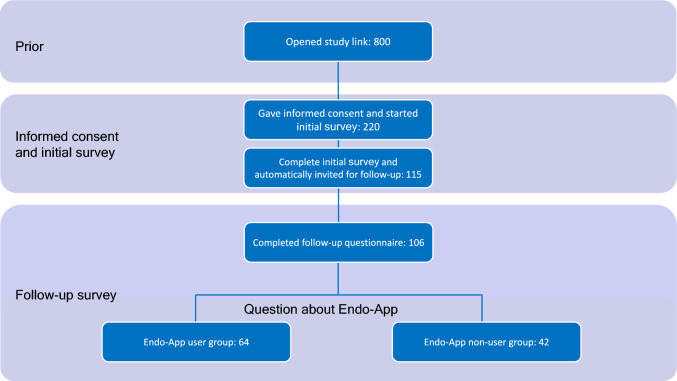
Patient flow

The sample consisted of 106 women. Inclusion criteria were a minimum age of 18 years, prior diagnosis of endometriosis and residency in Germany. Women were recruited through self-help groups, advertising on social media and posting in endometriosis-related groups. Women with endometriosis were invited to take part in the survey. Individuals who did not have endometriosis as a diagnosis or did not return both questionnaires were excluded from the study population (Fig. 1).

In the final sample, women’s age ranged from 20 to 51 years (mean: 33 years). Various occupational backgrounds were identified. Employees were most frequently represented (68 women), followed by students (14 women).

### Study design

Our primary goal with this study was to provide preliminary data on the potential efficacy and feasibility of the Endo-App in improving the quality of life for women with endometriosis. We selected the 2-week interval as it allowed us to give an initial rapid assessment and determine the immediate impact of the app on the participants’ quality of life.

The study consisted of an initial survey and a follow-up survey. The surveys were conducted using online questionnaires. The follow-up survey took place 2 weeks after the initial survey. The results of both questionnaires could be merged using participant coding while maintaining anonymity.

At the beginning of the questionnaire, all women were informed about the aims of the study, the anonymity of their data, and the contact details of the medical management for questions. Informed consent then was obtained.

### Initial survey

At the beginning of the questionnaire, screening criteria were collected (gender, age, diseases). In the next part of the survey, the women were asked about their quality of life. For this purpose, the standardized and validated Endometriosis Health Profile (EHP) questionnaire from Oxford University [18] was used. The implementation of the online questionnaire was reviewed by an Oxford University expert and found to be correct. The questionnaire asked about the five EHP core domains of quality of life:

- Pain (11 questions),
- Control and powerlessness (6 questions),
- Emotional well-being (6 questions),
- Social support (4 questions) and
- Self-image (3 questions).

In addition, two supplementary domains of the EHP were queried:

- Work life (5 questions) and
- Treatment (3 questions).

Participants were then asked to indicate their satisfaction with their own lives on a scale of 0 to 10 (quality of life index).

In the next section, women were asked to react to various statements concerning their endometriosis care. In addition, the women were asked about the impact of endometriosis on their work. The therapy used so far was also recorded.

In the following section, the women were asked about their menstrual periods. They were asked about hormonal therapy and the date of the first day of the last menstrual period to rule out confounding due to the overrepresentation of one cyclic phase.

In the last section of the questionnaire, the Endo-App was introduced and the women received information about the possibility to use the Endo-App free of charge.

### Follow-up survey after 2 weeks

Two weeks after the initial survey, women who had signed up for the mailing list were invited via email to complete the follow-up survey.

At the beginning of the follow-up survey, the women were asked about their usage behavior and intensity of use of the Endo-App. If women indicated that they had not used the Endo-App, they could voluntarily provide reasons.

The same domains of the EHP, quality of life index, and endometriosis care statements were queried for all women.

Additionally, for participants who indicated they had used the Endo-App, a section followed in which the women could evaluate individual features of the app.

### Recruitment and evaluation

Recruitment for this study was conducted online through various tools such as Facebook and Google. Online recruitment was deemed adequate to target women who are familiar with and actively use digital media.

By clicking on the respective link, participants were then taken to the online questionnaire, which was based on the SoSci Survey tool. The questionnaire could be completed either on a desktop computer or on a mobile device.

All women who participated in the first questionnaire could take part in a lottery for 10 € online vouchers, regardless if they entered their Email address for the second survey or not.

Only women who participated in both the initial survey and the follow-up survey were included in the analysis.

In the user group, the women who were evaluated (*n* = 64) were those who reported using the app. The women who participated both times, but indicated that they had not used the app (*n* = 42) were evaluated separately. These acted as a comparison group with which the user group could be compared to exclude an influence of general events, e.g. world events or weather, on the quality of life.

### Endometriosis Health Profile (EHP)

Each item of the EHP consists of a statement about the impact of endometriosis on the life of the affected person. The validated German survey was used. Responses are scaled from 0 = Never to 4 = Always. A domain’s EHP score is calculated from the average of the domain’s responses, scaled from 0 to 100, with a score of 0 being the best possible quality of life and 100 being the worst possible quality of life.

In addition to the EHP score per domain (corresponding to the mean of the items assigned to this domain), a summary “EHP-30 Summary Index” can also be formed. This is calculated as an equally weighted mean of the five core domains. The calculation procedure of the Summary Index was also applied to the “All Domains” evaluation so that an overall index could be created. This overall index including the additional domains has not been validated in the Oxford EHP-30. However, before calculating the overall index, the corresponding Cronbach’s alpha value was calculated to check the reliability of the scale and thus the feasibility of the index.

### Inferential statistics

To examine the change after two weeks of Endo-App use, statistical tests were performed with the variables described above.

First, an *F*-test was used to see if a *T*-test could be applied with equal variance. Then, if possible, a two-tailed paired *T* test was performed for each variable and significance was calculated. Furthermore, the effect size was calculated via Cohen’s *d*.

As a between-group comparison, pairwise group comparisons were performed for the Summary Index Change score using nonparametric two-sided Mann–Whitney *U* tests for unrelated samples.

Just as described in the EHP manual, this study also found a sufficiently large Cronbach’s alpha for the Summary Index (0.81) to consider it a summary index of the integrated individual domains.

A two-tailed, unpaired Mann–Whitney *U* test was used to compare the effects on the change scores of the Summary Index of the user group with a non-user group.

## Results

### Demographics

Table 1 shows a summary of the demographic characteristics of the participants. All participants were female and reported being diagnosed with endometriosis. The age ranged from 20 to 51 years. The average age of the user group was 33, the non-user group was 32.

**Table 1:** Demographic characteristics of the participants

	Users	Non-users	Total
Sex			
Female	64	42	106
Male	0	0	0
Diverse	0	0	0
Age			
Mean	33	32	33
Minimum	20	22	20
Maximum	51	46	51
Occupation			
High school student	0	0	0
Apprentice	2	3	5
University student	9	5	14
Employee	42	26	68
Public official	1	2	3
Self-employed	0	3	3
Unemployed	3	1	4
Other	7	2	9
Diagnoses			
Endometriosis	64	42	106
Breast cancer	0	0	0
History of infection with chlamydia trachomatis	2	2	4
Ovarian cancer	0	0	0
Fibroids	10	4	14
Smartphone			
Android	43		43
Apple iOS	21		21

Of the 106 participants, 64 self-reported using the Endo-App (user group). In contrast, 42 indicated that they had not used the Endo-App (non-user group). *N* = 15 women gave a reason for not using the app. Reasons fell into the categories of technical problems and little time for cell phone use.

Previous therapies mentioned were surgery (89%), hormone therapy (77%), endometriosis center consultation (59%), treatment by a gynecologist (78%), endometriosis rehabilitation (23%), support group (23%), period tracking apps (46%), alternative medicine (39%), and complementary therapies (41%). 42% used hormone therapy in a long-term cycle.

### Changes in the user group

An inferential statistical analysis was performed for the user group. This involved comparing the change in variables from initial to follow-up surveys. The results of the analysis are shown in Table 2.

**Table 2:** Comparison of variables before and during use of the Endo-App in the user group (*n* = number

		Initial survey		Follow up		Change				
Variable	*n*	M	SD	M	SD	M	T Stat	*p*		Cohen’s *d*
EHP										
Pain	64	52.66	17.95	43.43	21.39	− 9.23	− 5.80	0.0000	**	− 0.73
Control and powerlessness	64	70.51	22.37	55.92	23.54	− 14.58	− 6.15	0.0000	**	− 0.77
Emotional well-being	64	60.48	17.86	52.86	21.30	− 7.62	− 4.07	0.0001	**	− 0.51
Social support	64	64.75	23.92	54.69	29.53	− 10.06	− 3.81	0.0003	**	− 0.48
Self-image	64	55.86	27.08	51.30	29.96	− 4.56	− 1.66	0.1027		− 0.21
Work life	46	43.37	25.51	33.43	24.34	− 9.93	− 3.05	0.0038	**	− 0.45
Treatment	45	58.33	26.01	49.83	25.06	− 8.51	− 3.30	0.0019	**	− 0.49
*All Domains*	64	58.43	18.44	49.24	20.47	− 9.18	− 5.50	0.0000	**	− 0.69
*Summary Index*	64	60.85	17.72	51.64	21.02	− 9.21	− 5.38	0.0000	**	− 0.67
QoL (one-item)	64	4.94	1.97	5.63	1.55	0.69	3.80	0.0003	**	0.48

*M* median, *SD* standard deviation, *T Stat* = , *p* probability, *EHP* endometriosis health profile, *QoL* quality of life, ** change statistically significant

With regard to the EHP, an improvement was observed in all domains except *self-image*. The improvement of all variables except *self-image* was statistically highly significant. In the core domains *pain, control & powerlessness, emotional well-being, social support* and *self-image* all data sets were complete. In the *work life* and *treatment* modular questionnaires, 18 and 19 women, respectively, selected the “not applicable” response option offered in the modular questionnaire. All other questionnaires were complete.

For the variables *pain, control & powerlessness, emotional well-being, all domains* and summary index a strong effect could be found according to Cohen’s *d* (|*d*|> 0.5). Since *all domains* is not externally validated index, the Cronbach alpha value was calculated. This was *α* = 0.832, so the index could be included.

### Changes in the non-user group

The same inferential statistical analysis was performed for the non-user group. The results are shown in Table 3. There was a statistically significant change (improvement) in *pain* for the non-user group. There were no other significant results.

**Table 3:** Comparison of variables in the non-user group

		Initial survey		Follow up		Change				
Variable	*n*	M	SD	M	SD	M	T Stat	*p*		Cohens *d*
EHP										
Pain	42	42.80	22.55	37.12	22.39	− 5.68	− 3.38	0.0016	**	− 0.52
Control and powerlessness	42	54.66	24.83	52.28	24.59	− 2.38	− 0.83	0.4097		− 0.13
Emotional well-being	42	49.50	22.97	47.72	23.05	− 1.79	− 0.70	0.4861		− 0.11
Social support	42	48.66	26.32	50.30	24.69	1.64	0.43	0.6668		0.07
Self-image	42	39.48	28.64	38.69	26.08	− 0.79	− 0.30	0.7657		− 0.05
Work life	33	29.86	24.65	28.71	26.91	− 1.15	− 1.40	0.1704		− 0.24
Treatment	29	44.29	25.94	44.89	29.40	0.61	− 0.08	0.9380		− 0.01
*All Domains*	42	44.54	19.56	43.16	20.61	− 1.38	− 1.01	0.3200		− 0.16
*Summary Index*	42	47.02	20.74	45.22	21.00	− 1.80	− 0.88	0.3864		− 0.14
QoL (one-item)	42	6.10	1.46	6.31	1.63	0.21	1.32	0.1927		0.20

*n* number, *M* median, *SD* standard deviation, *T Stat* = ; *p* probability, *EHP* endometriosis health profile, *QoL* quality of life, ** change statistically significant

### Comparison of user group and non-user group

Table 4 compares the results of the user group and non-user group. ∆*M* represents the average change in the variables between the initial and follow-up surveys (see “change” columns in Tables 2 and 3). A direct comparison is not calculated in the table.

**Table 4:** Comparison of results between user group and non-user group

	User group				Non-user group			
Variable	*n*	∆*M*	*p*		*n*	∆*M*	*p*	
**EHP**								
Pain	64	− 9.23	0.0000	**	42	− 5.68	0.0016	**
Control and powerlessness	64	− 14.58	0.0000	**	42	− 2.38	0.4097	
Emotional well-being	64	− 7.62	0.0001	**	42	− 1.79	0.4861	
Social support	64	− 10.06	0.0003	**	42	1.64	0.6668	
Self-image	64	− 4.56	0.1027		42	− 0.79	0.7657	
Work life	46	− 9.46	0.0038	**	33	− 3.79	0.1704	
Treatment	45	− 9.81	0.0019	**	29	− 0.29	0.9380	
All Domains	64	− 9.06	0.0000	**	42	− 1.98	0.3200	
Summary Index	64	− 9.21	0.0000	**	42	− 1.80	0.3864	
**QoL (one-item)**	64	0.69	0.0003	**	42	0.21	0.1927	

*n* number, *∆M* change of median, *p* probability, *EHP* endometriosis health profile, *QoL* quality of life, ** change statistically significant

To test whether the difference between the user group and non-user group for the summary index was significant, a two-sided Mann–Whitney *U* test was calculated for the summary index. This was highly significant with *p*-value = 0.006477.

### Days since the beginning of the last menstrual period

Table 5 provides an overview of the phase of the menstrual period at the time of the initial survey. The purpose of analyzing the menstrual periods was to exclude confounding. For this purpose, the time span between the first day of the last menstrual period and the date of participation in the survey was calculated. The cycle-point was then grouped into weeks. Participants in the long-term cycle were recorded separately, as no meaningful date of the last menstrual period could be determined for them.

**Table 5:** Days since last menstrual period (*n* = number)

	Users		Non-users	
Days since last period	*n*	Proportion	*n*	Proportion
0–7	9	13%	7	13%
8–14	10	14%	10	19%
14–21	9	13%	3	6%
22–28	9	13%	3	6%
> 28	9	13%	8	15%
Long cycle	25	35%	22	42%

## Discussion

In this study already after two weeks of use, a statistically significant improvement in quality of life was observed among Endo-App users both according to EHP and the quality of life index. There was a comparable age structure and demographic structure in both groups to other endometriosis studies. In a large-scale Swedish study on endometriosis the participants were considerably older with a mean age of 36.7 years and an age range between 16 and 67 years [19]. Although there was a slight decrease in symptoms with age, various endometriosis symptoms, reduced health-related quality of life, and limitations in daily living were present in the entire study population. Quality of life according to the summary index improved by −9.21 points. This coincided with the improvement according to the quality of life scale of 0.69 points.

It is noteworthy that the Endo-App did not have an equal impact on all domains of the EHP quality of life.

After two weeks of use, the impact of endometriosis on work life was reduced by an average of −9.93 points. Likewise, the variable for control and helplessness improved by −14.58 points. There was also an improvement of −9.23 points in the pain domain. Only the domain self-image resulted in merely a small improvement of −4.56 points, which was not statistically significant.

To exclude bias in quality of life scores due to increased symptoms during the menstrual period, the timing of the menstrual cycle was determined at the time of the initial survey. An accumulation of the same menstrual cycle phase, for example, day 1–7 at the time of the initial survey, could theoretically lead to an over- or underestimation of the effect. There was no significant clustering of menstrual cycle phases among participants during the initial or follow-up surveys and an even distribution across all menstrual cycle phases and women on long-cycle hormone therapy (see Table 5). Consequently, it can be assumed that there was no significant bias in the results due to specific menstrual phases.

Analogous to a bias due to the menstrual phase, it would be possible for the results to be positively influenced by external influences. Such influences could be, for example, changes in the seasons or societal changes such as changes in corona measures. To test this, the results of women who used the Endo-App were compared with those who did not use the Endo-App. Because of the observational design, the study did not meet the requirements for a control group in an interventional study. For example, no randomization was performed. Nevertheless, the participants, who according to their own statements did not use the Endo-App, offered themselves as a point of comparison. The comparison was shown in the results section in Table 4.

In the user group, nine out of ten variables were improved in a highly significant manner.

In the summary index, the observed changes after two weeks regarding the user group and non-user group differed by −7.41. The comparison of changes between the user group and the non-user group on the summary index was validated with a Mann–Whitney *U* test. The result of the test was highly significant (*p*-value = 0.006477). This confirms the hypothesis that a corresponding improvement in quality of life was triggered by the Endo-App.

Overall, the comparison with the non-user group supports the hypothesis that the Endo-App itself is responsible for the improvement in quality of life and that this is not due to external influences.

It would also be theoretically possible that the non-user group did not use the Endo-App because of substantial deterioration in their quality of life. In this case, the quality of life scores between groups would be biased by systematic self-selection. To exclude this possibility, reasons for non-use were recorded and subjected to careful scrutiny in the analysis. The reason for non-use was recorded via free text in the follow-up survey. 15 of the 42 non-users surveyed gave a reason in the free text. In 14 cases, the reason was based on technical problems during installation. These technical difficulties can be explained by the fact that the app was made available via Apple Testflight and Android APK, which was unfamiliar to some users and thus represented a technical challenge. Since the occurrence of technical difficulties is in no way related to the quality of life or endometriosis symptoms of those affected, it can be assumed that the classification of the women into the non-user group did not result in any relevant distortions in terms of content.

The time interval between the initial interview and the follow-up interview may already display short-term changes with the Endo-App. Based on the current literature, it can be expected that the effect would also be detectable after a longer period of time [14, 20, 21]. This needs to be the subject of future longitudinal studies with longer survey periods.

It is also expected that some of the Endo-App content would reach its maximum effect over a longer period of use. For example, physiotherapy exercises, yoga, mindfulness exercises, or a healthier diet have short-term effects but also build up their effect over several weeks and months [22, 23] and maintain this effect in the long term [24]. Hansen et al. were able to show that even six years after an intervention with mindfulness and acceptance and commitment therapy, the improvement achieved was still detectable [25].

It is noteworthy that the survey with the EHP always looks at the last four weeks in the women’s lives, because the items of the questionnaire are designed for this period and, according to Oxford, may not be changed. This means that at the follow-up survey, two of the four weeks in the period under consideration were still prior to the use of the Endo-App. This in turn suggests that even stronger effects or improvements would be observable in a follow-up survey that would take place longer after the initial survey.

The results presented here allow initial conclusions to be drawn regarding the beneficial effect of the Endo-App. However, it must be pointed out that there are limitations in the study design.

The analyses shown were not adjusted for multiple testing. Thus, across all hypotheses tested, there is a greater risk of alpha error than 5%. It should be noted, however, that changes in many domains and especially the summary index of the user group remain significant even after adjustment for multiple testing following the Bonferroni-Holm method.

Another limitation is the rather small sample size, although it was large enough to achieve sufficient power for finding significant effects. However, testing the effects found here with a higher number of participants is an important area for further studies on the topic.

The endometriosis diagnosis was recorded by self-reporting. In this context, Shafrir and colleagues conclude in a review article that endometriosis can be validly assessed by self-report [26]. When study participants indicate a surgical diagnosis of their endometriosis in the questionnaire, there is over 94% agreement with medical records. This supports the validity of the predominantly surgical diagnoses reported in the study. Nevertheless, the endometriosis diagnosis is important, which is why it should be assessed and verified in more detail in subsequent studies.

The following studies should also include details about comorbidities and past treatments. It could also be considered including rASRM and ENZIAN classifications, although they do not provide information about symptom intensity.

Although complete case analysis is often used in medical studies [27] it has its limits, especially when the reason for dropouts is not known [28]. For the necessary randomized trial measures to prevent dropouts like reminders and recruitment via medical personnel should be implemented as well as investigations concerning the dropout reason.

Despite the limitations, the results found are an important starting point to plan a randomized controlled trial subsequently and to support the exploration of expected effects. The results also clearly point to the meaningfulness and usefulness of such a follow-up study due to the expected positive effects.

## Conclusion

In this observational pilot study, it could be shown that a significant improvement in the quality of life occurred in the surveyed users of the Endo-App already after 2 weeks. This included several relevant domains of quality of life and could not be explained by external or confounding variables. As the used questionnaire is validated specifically for endometriosis, the effects shown in this study show an effectiveness specifically for women with endometriosis.

However, further research on the Endo-App requires a randomized controlled trial with sufficient power to provide confirmatory insights into the medium- and long-term effects.

In summary, the results are very promising, so it is expected that women with endometriosis will benefit from the use of the Endo-App. The Endo-App can help to implement multimodal self-help measures in the daily life of endometriosis patients through various functions such as symptom diary and interactive exercises. Considering the substantial burden of endometriosis and the identified gaps in patient care, further investigation is of great interest.

## Data Availability

Original data is available upon reasonable request from the authors.
